# Multi-provincial Salmonellosis Outbreak Related to Newly Hatched Chicks and Poults: A Genomics Perspective

**DOI:** 10.1371/currents.outbreaks.309af53b9edcc785163539c30c3953f6

**Published:** 2017-08-09

**Authors:** Matthew A. Croxen, Kimberley A. Macdonald, Matthew Walker, Nancy deWith, Erin Zabek, Christy Peterson, Aleisha Reimer, Linda Chui, Lorelee Tschetter, Linda Hoang, Robin K King

**Affiliations:** British Columbia Centre for Disease Control Public Health Laboratory, Vancouver, British Columbia, Canada; National Microbiology Laboratory (NML), Winnipeg, Manitoba, Canada; British Columbia Centre for Disease Control (BCCDC) Public Health Laboratory, Vancouver, British Columbia, Canada; Public Health Agency of Canada, Canadian Science Center for Human and Animal Health, Winnipeg, Manitoba, Canada; Animal Health Branch, BC Ministry of Agriculture, Abbotsford, British Columbia, Canada; Animal Health Centre, BC Ministry of Agriculture, Abbotsford, British Columbia, Canada; Public Health Agency of Canada, Winnipeg, Manitoba, Canada; Department of Public Health Genomics, Public Health Agency of Canada, National Microbiology Laboratory, Winnipeg, Manitoba, Canada; Laboratory Medicine and Pathology, University of Alberta, Edmonton, Alberta, Canada; PulseNet Canada, Public Health Agency of Canada, Winnipeg, Manitoba, Canada; British Columbia Centre for Disease Control, Vancouver, British Columbia, Canada; Alberta Agriculture and Forestry, Government of Alberta, Edmonton, Alberta, Canada

## Abstract

**Background:**

A multi-provincial outbreak of Salmonella enterica serovar Enteritidis was linked to newly hatched chicks and poults from a single hatchery during the spring of 2015. In total, there were 61 human cases that were epidemiologically confirmed to be linked to the chicks and poults and the outbreak was deemed to have ended in the summer of 2015.

**Methods:**

PulseNet Canada, in coordination with the affected provinces, used genome sequencing of human and agricultural Salmonella Enteritidis isolates to aid in the epidemiological investigation, while also using traditional typing methods such as phagetyping and pulsed-field gel electrophoresis (PFGE).

**Results:**

All human outbreak cases, except one, were Phage Type (PT) 13a. Single nucleotide variant analysis (SNV) was able to provide a level of resolution commensurate with the results of the epidemiological investigation. SNV analysis was also able to separate PT13a outbreak-related isolates from isolates not linked to chicks or poults, while clustering some non-PT13a agricultural strains with the outbreak cluster.

**Conclusions:**

Based on conventional typing methods (phagetyping or PFGE), clinical and agricultural PT13a SE isolates would have been considered as part of a related cluster. In contrast, phagetyping would have led to the exclusion of several non- PT13a strains that clustered with the outbreak isolates using the genome sequence data. This study demonstrates the improved resolution of genome sequence analysis for coordinated surveillance and source attribution of both human and agricultural SE isolates.

## Introduction

Salmonellosis is one of the most common enteric foodborne illnesses reported in Canada. The Public Health Agency of Canada estimated the annual incidence of *Salmonella enterica* related foodborne illness at approximately 269 cases per 100,000 population^1^. In Canada, *Salmonella enterica* serovar Enteritidis (SE) is the most common laboratory confirmed serotype found in cases of human salmonellosis^2^. Phagetypes (PT) 8, 13, and 13a make up the majority of domestically acquired human cases, while the same PTs are also predominant in chickens^3^. Not surprisingly, *Salmonella* places a major burden on health, economic and agricultural systems.

Recently, the United States has reported a number of multi-jurisdictional *Salmonella* (various serotypes) outbreaks linked to live poultry from mail-order hatcheries^4^^,^^5^^,^^6^^,^^7^ and backyard flocks^6^^,^^7^, including an epidemiological review of 53 outbreaks related to live poultry from 1990 through 2014^8^. Starting in the spring of 2015, several Canadian provinces experienced outbreaks of SE that were traced back, epidemiologically, to the sale of recently hatched chicks and poults from a single hatchery. A total of 61 SE-related human illnesses were reported from the provinces of Alberta, British Columbia, Manitoba, the Northwest Territories and Saskatchewan, between January and June 2015. While Canada has several different surveillance systems for *Salmonella* that captures investigations from human, food, and environmental isolates, it has traditionally been difficult to link isolates due to the limited resolution provided by traditional methods such as pulsed-field gel electrophoresis (PFGE) and phagetyping (PT). Historically, SE isolates from agricultural sources have rarely been included in surveillance activities, creating a major gap in data acquisition. As PulseNet Canada modernizes its surveillance system to use genome sequencing of enteric pathogens, this outbreak was an opportunity to use a high-resolution typing method, single nucleotide variant (SNV) analysis, of SE isolates from multi-provincial clinical and agricultural sources to corroborate the epidemiological investigation. Analysis of genome sequencing data has previously been demonstrated for surveillance and outbreaks of several *Salmonella* serovars in Canada^9^ , United States^10^, Australia^11^ and the UK^12^^,^^13^. The SNV analysis in this study was done in conjunction with federal epidemiologists to support the outbreak investigation. Much like the international effort published by Public Health England, and their tracking of SE in an egg distribution network, this example further demonstrates the utility of using genome sequencing for outbreak investigation and source attribution.

## Methods

***Microbiology***

All clinical SE isolates were recovered at provincial public health laboratories from Alberta, British Columbia, Manitoba, New Brunswick, Newfoundland, Prince Edward Island, Quebec, and Saskatchewan. Agricultural isolates were provided by the British Columbia Ministry of Agriculture Animal Health Centre (BC MAgri) and Alberta Agriculture and Forestry (AAF). *Salmonella* was identified using standard biochemical assays, and serotyped following the Kauffmann-White scheme^14^^,^^15^. Phagetyping (PT) was performed at the Public Health Agency of Canada National Microbiology Laboratory (PHAC-NML) in Winnipeg and Guelph. PFGE was performed using PulseNet’s standard operating procedure^16^ on human and agricultural SE PT13a isolates received between January and June 2015 at provincial public health laboratories, AAF Agri-Food Laboratories, PHAC-NML, and PHAC-NML-Guelph. Non-PT13a isolates were included where one or both of the XbaI/BlnI PFGE patterns matched the dominant PT13a PFGE patterns (i.e., SENXAI.0006 and/or SENBNI.0007).

***Genome Sequencing***

Genomic DNA was isolated from the SE isolates using the Qiagen DNeasy Blood & Tissue kit (Mississauga, Ontario) and sequencing libraries were generated using the Illumina Nextera XT library preparation kit (San Diego, California). The samples were sequenced by the Genomics Unit at the PHAC-NML using a 2 x 300 bp (600 cycle) Illumina MiSeq Reagent Kit v3 on an Illumina MiSeq. All sequences are available at NCBI under BioProject PRJNA343897.

***Single Nucleotide Variant Calling***

The Single Nucleotide Variant Phylogenomics (SNVPhyl) pipeline^17^, designed by the NML bioinformatics team, was used to call single nucleotide variants (SNV) using a SPAdes^18^ assembled (closed by Sanger sequencing) BC SE isolate 08-3405 (PT8; PFGE SENXAI.0003/SENBNI.003) as the reference genome. The phylogenetic tree was generated using plotTree.py^19^, a wrapper script for the ETE toolkit^20^.

## Results and Discussion

PHAC-NML sequenced SE isolated from clinical cases that had occurred between January and June 2015 and that had PFGE-Xbal pattern SENXAI.0006. Agricultural SE isolates that coincided with the clinical investigation from hatchery samples and trace-out farms with PFGE profiles SENXAI.0006/SENBNI.0007 were forwarded to PHAC-NML for sequencing, regardless of phagetype. SE isolates from trace-back samples from backyard flock owners were sent directly to PHAC-NML for sequencing without PFGE characterization. PFGE using a second enzyme, BlnI, was completed on 492 of 574 isolates, and the majority exhibited the SENBNI.0007 pattern (477 of 492 isolates). Of the 574 SE isolates in the study, 527 were PT13a, with the remaining 47 isolates typed as PT8, PT13, PT19, PT22, PT23, ATEN-83 or atypical. Moreover, of the 47 non-PT13a SE isolates, 39 shared the same SENXAI.0006 PFGE pattern as 520 of the PT13a SE isolates, demonstrating the obfuscation of using PFGE or PT to rule in or rule out epidemiological linkages.

In total, 575 SE isolates were sequenced (574 plus the reference 08-3405) on the Illumina MiSeq. Of these sequenced SE isolates, 149 were clinical isolates (including 08-3405 reference) and 426 were agricultural isolates (AAF: 226; BC MAgri: 200). Sixty of the 149 sequenced clinical SE isolates had confirmed exposure to chicks or poults (Alberta: 35; British Columbia: 18; Manitoba: 1; and Saskatchewan: 6). Not all confirmed clinical cases from the epidemiological investigation were sequenced.

The distribution of clinical isolates is shown in Figure 1.

**Distribution across provinces of clinical SE isolates sequenced in this study.**
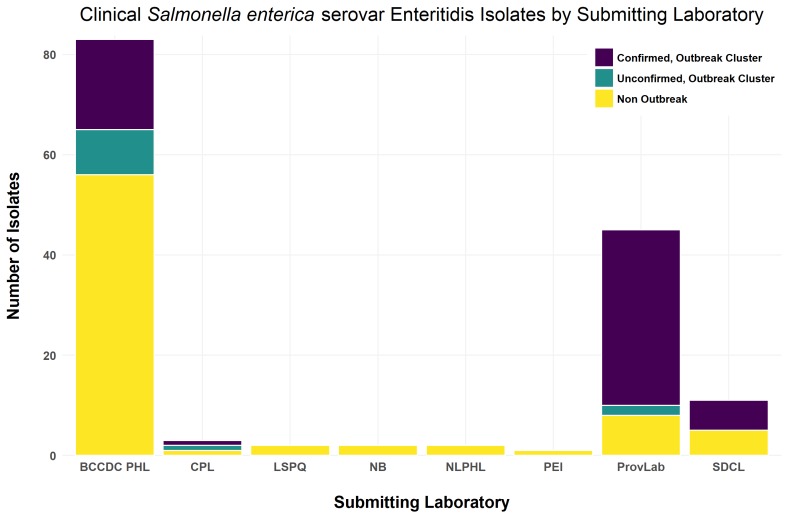
Isolates are grouped based upon submitting laboratories. BCCDC PHL (BC Centre for Disease Control Public Health Laboratory; British Columbia), CPL (Cadham Provincial Laboratory; Manitoba), LSPQ (Laboratoire de santé publique du Québec, Québec), NB (New Brunswick), NLPHL (Newfoundland & Labrador Public Health Laboratory; Newfoundland), PEI (Prince Edward Island), ProvLab (Provincial Laboratory for Public Health, Alberta), SDCL (Saskatchewan Disease Control Laboratory, Saskatchewan)

The sequenced isolates were clustered using the SNVPhyl pipeline, which identified 441 high quality SNVs (hqSNVs) for phylogenetic reconstruction (Figure 2).

**Maximum-likelihood phylogenetic inference of outbreak and non-outbreak SE isolates.**
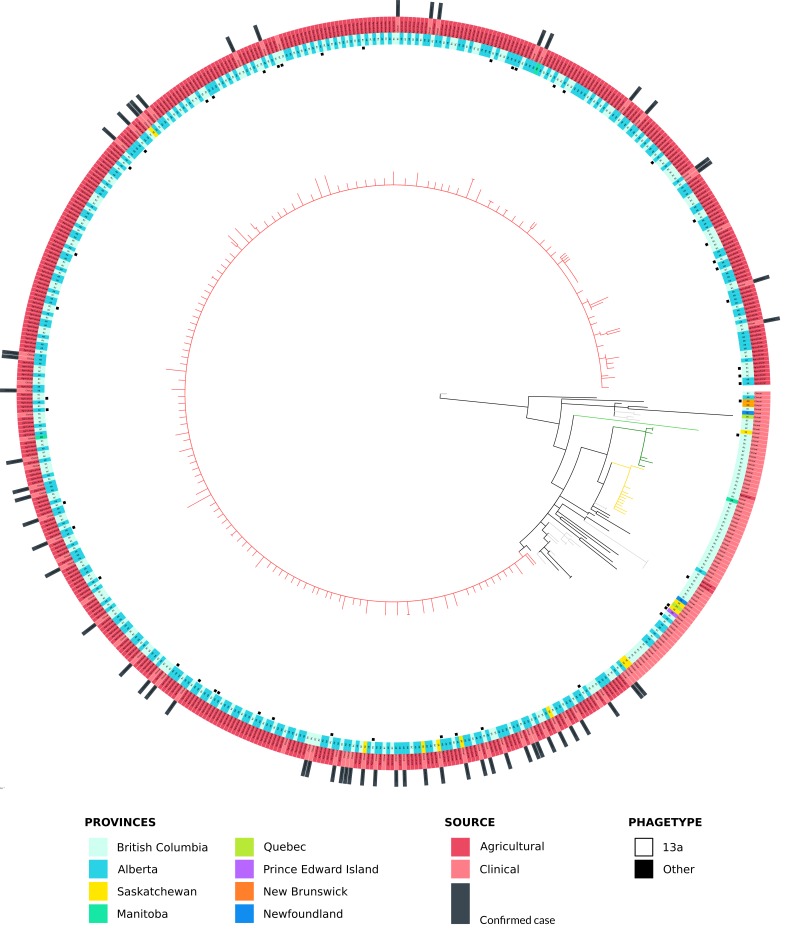
SNVPhyl was used to map all of the SE isolates against the 08-3405 reference genome (bright green) and call high quality SNVs (hqSNV) from the core genome. Over 85% of the sequence data mapped to the reference genome and a total of 441 core hqSNVs were used to construct the phylogenetic tree. The outbreak clade is shown as red branches (note that the outbreak isolates 15-4336 and 15-4529 are not represented in this tree), while two other uninvestigated clades are shown in gold and dark green branches. Smaller clusters are highlighted as light grey branches. The inner most ring indicates non-PT13a phagetypes; if no black square is present then the isolate is PT13a. The second inner most ring (mainly cyan and turquoise) indicates the province where the isolate originated. The second outer-most ring (shades of red) indicates if the isolates came from a clinical or agricultural source. Finally, the dark grey bars in the outer-most ring indicate cases with confirmed exposure to chicks and poults.

The SE genomes from the epidemiologically confirmed cases (dark grey lines, Figure 2) clustered in one large clade called the outbreak clade (red branches, Figure 2). This outbreak clade contains 493 of the sequenced isolates, including clinical isolates from Alberta, British Columbia, Manitoba, and Saskatchewan, as well as SE isolates collected by the BC MAgri and AAF. There was a maximum of 8 hqSNV differences between the 493 isolates (range 0 – 8 SNVs). While the majority of SE isolates were PT13a, 42 isolates were non-PT13a (black square in Figure 2), suggesting that SE phagetype results may not be sufficiently discriminatory to exclude an isolate from epidemiological investigation. This is supported by technical challenges and limitations of *Salmonella* phagetyping^21^, compounded by biological changes that can lead to phagetype conversion^22^. During the clustering of all of the SE isolates, two additional clades outside of the outbreak clade emerged. The first clade (gold branches, Figure 2) consisted of 18 clinical SE isolates from British Columbia and Manitoba. The second clade (green branches, Figure 2) was made up of 15 BC SE isolates, 1 of which was collected from BC MAgri. These two clusters were not followed up epidemiologically. These omissions illustrate the ability of genomics-based methods to identify potential linkages of clinical isolates to other provinces or sectors, such as agriculture, and highlight the need for improved surveillance methods based on better isolate characterization.

Sixty-one human cases [Alberta (35), British Columbia (19), Manitoba (1), NWT (1), and Saskatchewan (5)], were linked to the outbreak by epidemiological investigation^23^; 58 of these isolates were sequenced in this study. All patients recalled contact with live chicks or poults^23^. The SNV-based analysis also clustered 12 clinical isolates with the outbreak clade that were either unconfirmed or were not included in the epidemiological investigation (Figure 2). This highlights the added value of performing real-time genome sequencing on a routine basis in reference laboratories, as SNV-based clustering of *Salmonella* genome sequences can better rule-out relationships more accurately than traditional methods (e.g., PFGE and PT). The ability to include and exclude isolates through SNV-based clustering can provide better targeted data for epidemiological investigations, increase the likelihood of finding a source and in a timelier fashion, and provide a more effective prevention strategy to reduce illness and economic loss.

Illnesses associated with the implicated hatchery exemplifies how salmonellosis causes a high economic burden of illness on the health-care system, and as well, on the agricultural industry and the economy as a whole. This burden will continue unless better discriminatory techniques are applied to clinical and agricultural SE isolates to promote better source tracking, and enable targeted mitigation actions and prevention of food-borne illness. Integrated surveillance to capture the full farm-to-fork spectrum of SE is critical in providing a scientific base for source attribution, informing policies and intervention/prevention strategies, and ultimately reducing financial burdens on the health care, food and agricultural systems.

Contact with live poultry was linked to over 500 cases of salmonellosis **in the US in 2013 and 2014^4^^,^^5^, and during the following two years, over 1,100 salmonellosis cases were **linked to backyard flocks^6^^,^^7^. Routine genome sequencing of *Salmonella* by Public Health England (BioProject PRJNA248792) Centers for Disease Control and Prevention's PulseNet USA (BioProject PRJNA230403)^13^, and the United States Food and Drug Administration’s GenomeTrakr (BioProject PRJNA183844)^24^ sets a new standard with a model for genomic data sharing that also provides a wealth of information for international surveillance. Given the time frame of the Canadian outbreak in 2015, comparisons of the deposited SE sequences in GenomeTrakr during these concurrent outbreaks linked to live poultry^6^ could have provided an opportunity to compare our SE outbreak isolates with those collected south of the border. Successful international source-attribution was exemplified by Public Health England, linking SE cases internationally to eggs – actions all driven by analysis of genome sequence data^12^. Given this framework, it is clear that genome sequencing can provide source attribution of clinical isolates as the current gold standard for rapid response and preventive public health activities. It is also the new gold standard for source attribution and pan-system collaborations. We believe that genome sequencing-based surveillance standardized at the national and international level, will provide the foundation to begin to fill the knowledge gap between SE illness in the human population and source attribution in the agriculture, and food sectors.

Detection of foodborne illness by PulseNet USA was estimated to prevent approximately 270,000 cases of foodborne illness each year, saving $500 million in costs to the US economy^25^. As the PulseNet USA program is reported to cost $7.3 million per year, the cost of surveillance programs far outweighs the costs of uncontrolled outbreaks of foodborne illness. Although the estimated burden of foodborne illness to the Canadian economy is lower than that of the United States, salmonellosis still causes frequent outbreaks of foodborne illness each year in Canada and is associated with a high economic toll. For every reported case, there are an estimated 26 unreported cases in Canada^1^. Although these cases may not be the largest strain on health-care, they are still an unnecessary burden on a system that is cost-constrained and can result in loss of productivity and other economic costs associated with absence from work. Development of standardized methods for routine use of genome sequencing at the national and international levels, of other enteric pathogens should further improve on these SE successes. This approach is now being adopted by PulseNet USA, the Food and Drug Administration^24^, Public Health England^13^^,^^26^, and PulseNet Canada, among others worldwide.

## Conclusions

This was the first outbreak in Canada to provide direct evidence of how genome sequencing can perform source attribution and provide improved data for targeted outbreak investigations salmonellosis. This multi-discipline, multi-jurisdictional, and collaborative investigation also highlighted the importance of timely integrated surveillance based on the farm-to-fork continuum and a One Health concept. While this outbreak investigation was completed without genome sequence analysis, SNV-based clustering was effective for source-tracking of SE during an outbreak associated with exposure to live chicks and poults, while also identifying potentially related clinical isolates that were not epidemiologically investigated. Additionally, other clusters were identified that contained either multi-provincial SE isolates, or clinical isolates with agricultural isolates, illustrating the ability of genome sequencing to provide more information than PT or PFGE for epidemiological investigation. Genome sequencing in provincial public health laboratories on a routine basis has the potential for significant savings to the health care system, agricultural/food industries, and to the Canadian economy. Given the challenges and limitations of conventional typing methods such as PFGE and phagetyping, and the worldwide success and resolution of genome sequence analysis, PulseNet Canada will be ceasing phagetyping and PFGE and is moving towards genome sequencing as the gold standard for surveillance, outbreak investigations, and source attribution.

## Corresponding Author

Robin King, Alberta Agriculture and Forestry, Edmonton, Alberta. robin.k.king@gov.ab.ca

## Competing Interests

The authors have no competing interests to declare.

## Data Availability

All sequence data is available at NCBI under BioProject PRJNA343897. All corresponding data (e.g., PT, PFGE) can be found in the Supplementary Table available under doi:10.6084/m9.figshare.5007773.v1 on figshare.
